# Predicting the Need for Blood Transfusions in Cardiac Surgery: A Comparison between Machine Learning Algorithms and Established Risk Scores in the Brazilian Population

**DOI:** 10.21470/1678-9741-2023-0212

**Published:** 2024-02-21

**Authors:** Cristiano Berardo Carneiro da Cunha, Tiago Andrade Lima, Diogo Luiz de Magalhães Ferraz, Igor Tiago Correia Silva, Matheus Kennedy Dionisio Santiago, Gabrielle Ribeiro Sena, Verônica Soares Monteiro, Lívia Barbosa Andrade

**Affiliations:** 1 Department of Cardiovascular Research, Harvard Medical School, Boston, Massachusetts, United States of America; 2 Department of Cardiovascular Research, Brigham and Women’s Hospital, Boston, Massachusetts, United States of America; 3 Department of Cardiovascular Surgery, Instituto de Medicina Integral Professor Fernando Figueira (IMIP), Recife, Pernambuco, Brazil; 4 Department of Systems Analysis and Development, Instituto Federal de Pernambuco, Recife, Pernambuco, Brazil; 5 Department of Medicine, Faculdade Pernambucana de Saúde, Recife, Pernambuco, Brazil; 6 Department of Cardiology, Instituto de Medicina Integral Professor Fernando Figueira (IMIP), Recife, Pernambuco, Brazil; 7 Department of Post-Graduation, Instituto de Medicina Integral Professor Fernando Figueira (IMIP), Recife, Pernambuco, Brazil

**Keywords:** Blood Transfusion, Cardiac Surgery, Risk Prediction, Machine Learning

## Abstract

**Introduction:**

Blood transfusion is a common practice in cardiac surgery, despite its well-known negative effects. To mitigate blood transfusion-associated risks, identifying patients who are at higher risk of needing this procedure is crucial. Widely used risk scores to predict the need for blood transfusions have yielded unsatisfactory results when validated for the Brazilian population.

**Methods:**

In this retrospective study, machine learning (ML) algorithms were compared to predict the need for blood transfusions in a cohort of 495 cardiac surgery patients treated at a Brazilian reference service between 2019 and 2021. The performance of the models was evaluated using various metrics, including the area under the curve (AUC), and compared to the commonly used Transfusion Risk and Clinical Knowledge (TRACK) and Transfusion Risk Understanding Scoring Tool (TRUST) scoring systems.

**Results:**

The study found that the model had the highest performance, achieving an AUC of 0.7350 (confidence interval [CI]: 0.7203 to 0.7497). Importantly, all ML algorithms performed significantly better than the commonly used TRACK and TRUST scoring systems. TRACK had an AUC of 0.6757 (CI: 0.6609 to 0.6906), while TRUST had an AUC of 0.6622 (CI: 0.6473 to 0.6906).

**Conclusion:**

The findings of this study suggest that ML algorithms may offer a more accurate prediction of the need for blood transfusions than the traditional scoring systems and could enhance the accuracy of predicting blood transfusion requirements in cardiac surgery patients. Further research could focus on optimizing and refining ML algorithms to improve their accuracy and make them more suitable for clinical use.

**Table:** 

Abbreviations, Acronyms & Symbols				
AUC	= Area under the curve		ML	= Machine learning
BPT	= Blood prediction tool		MLP	= Multi-layer perceptron
BSA	= Body surface area		PI	= Permutation importance
CABG	= Coronary artery bypass grafting		RF	= Random forest
CI	= Confidence interval		ROC	= Receiver operating characteristic
COVID-19	= Coronavirus disease 2019		SD	= Standard deviation
CPB	= Cardiopulmonary bypass		SVM	= Support vector machine
Hb	= Hemoglobin		TRACK	= Transfusion Risk and Clinical Knowledge
LIME	= Local interpretable model-agnostic explanations		TRUST	= Transfusion Risk Understanding Scoring Tool
LR	= Logistic regression			

## INTRODUCTION

Blood transfusion is widely utilized in cardiac surgery to compensate for significant blood loss during operations. However, this procedure has well-documented adverse effects, including an increased risk of infection, transfusion-related acute lung injury, and transfusion-related immunomodulation^[1,2]^.The identification of patients at higher risk of requiring blood transfusions is crucial to prevent complications and optimize outcomes. By doing so, healthcare professionals can take proactive measures to prevent complications and optimize patient outcomes^[3,4]^. Furthermore, limited availability of blood products underscores the need for strategic preventive measures to manage the demand for transfusions and minimize their use when possible.

To evaluate the efficacy of existing blood transfusion predictive models, validation studies have been conducted across diverse patient populations addressing their inherent limitations^[5,6]^. One such study examined the widely used Transfusion Risk and Clinical Knowledge (TRACK) and Transfusion Risk Understanding Scoring Tool (TRUST) scoring systems, revealing their less-than-optimal accuracy when applied to specific patient cohorts^[7]^. This finding highlights the inadequacy and unreliability of these models for all patients and emphasize the need for further research and for the development of more precise and effective models to predict blood transfusion needs.

The accuracy limitations of the currently available scoring systems can be attributed to variations in patients’ demographics, clinical characteristics, and surgical practices across different populations^[7]^. Machine learning (ML) algorithms have the potential to offer more accurate predictions by analyzing complex interactions between patients’ characteristics and surgical factors^[8]^, making them a promising approach for improving the accuracy of blood transfusion prediction models. Thus, the objective of this study was to develop a personalized predictive model to assess blood transfusion risk in patients undergoing major cardiac surgery, using ML (blood prediction tool [BPT]).

## METHODS

This research study aims to evaluate the effectiveness of ML techniques in predicting blood transfusion requirements among a cohort of 495 patients who underwent cardiac surgery at the Department of Cardiology of Instituto de Medicina Integral Professor Fernando Figueira (or IMIP) (Pernambuco, Brazil) between the years 2019 and 2021. The blood transfusion protocol implemented at the institution follows a restrictive strategy based on bedside hemodynamic and gasometric parameters. According to this strategy, blood transfusion is recommended only when the hematocrit value falls < 24% from the initiation of surgery until discharge to the intensive care unit^[9]^. It is important to note that the service does not employ any equipment for the reuse of intraoperative blood. The study was approved by the ethics committee of the Instituto de Medicina Integral Professor Fernando Figueira (opinion number 5.259.262).

### Variables and Algorithm Selection

The dataset utilized in this study comprised various demographic factors, preoperative laboratory test results, comorbidities, and surgical characteristics, all of which are significant factors that could impact a patient’s surgery response and the required amount of blood during the operation.

The dataset was initially randomly divided into training (80%) and testing (20%) sets to ensure unbiased model evaluation. Feature selection was employed to identify the most significant variables for predicting blood transfusion requirements in cardiac surgery patients. Only statistically significant variables were included in the ML models. Categorical variables were then converted into numerical values to enable their utilization in the ML algorithms. Furthermore, to ensure consistent scaling and comparison of different features, all variables were normalized within the range of 0 to 1. Additionally, the training data was balanced using the Synthetic Minority Over-sampling Technique (or SMOTE)^[10]^ to address any potential class imbalance.

This study utilized four ML models, including support vector machine (SVM), random forest (RF), logistic regression (LR), and multi-layer perceptron (MLP), which have demonstrated exceptional performance in various medical domains, highlighting their effectiveness and versatility in healthcare applications^[11]^. LR was also employed for the calibration of the TRACK and TRUST scores, enhancing their accuracy. To optimize the models’ performance, Bayesian optimization was employed, intelligently exploring the hyperparameter space and identifying the optimal settings that maximize predictive capabilities. Stratified k-fold cross-validation^[12]^ was applied to ensure a robust evaluation of the models’ performance by dividing the data into representative folds with consistent class distributions.

To ensure a rigorous statistical analysis of the results, non-parametric tests, specifically the Wilcoxon-Mann-Whitney test, were employed due to the non-normal distribution of the data. Statistical significance was determined using a significance level of *P*<0.05. After identifying the best-performing algorithm, a permutation importance (PI)^[13]^ analysis was conducted to assess the relative importance of features. This technique involves randomly permuting the values of each feature and observing the resulting impact on the model’s performance, providing a quantitative evaluation of each feature’s contribution to the overall accuracy. PI is widely recognized as a robust method that directly measures the influence of features on the model’s performance. Also, local interpretable model-agnostic explanations (LIME) technique will be used, providing insights into how the tool considers all the features to make a prediction. LIME aims to provide local interpretability for complex predictive models by approximating them with simpler, interpretable models within localized regions of the input space^[14]^. By perturbing the input data and observing the resulting changes in the model’s predictions, LIME generates explanations that highlight the importance and contributions of each feature in the decision-making process, which is valuable in domains prioritizing interpretability and transparency.

The results are available, along with a link to the BPT tool, and can be accessed and used online at the website https://github.com/tiagopessoalima/bpt/tree/main.

## RESULTS

The association between patients’ features and the requirement for blood transfusion is presented in Table 1. Among the study participants, 284 individuals (57.4%) needed the administration of at least one bag of blood transfusion. The analysis revealed associations between blood transfusion and older age, smaller body surface area (BSA), lower hemoglobin levels, and being female. Additionally, a significant association was observed between blood transfusion and prior cardiac surgery and use of cardiopulmonary bypass (CPB). However, the presence of diabetes mellitus and high blood pressure did not exhibit a significant association with the need for blood transfusion. Furthermore, neither the urgency of the procedure nor the type of surgery performed demonstrated a significant relationship with the requirement for blood transfusion. Despite hematocrit’s statistical significance, its strong correlation (coefficient: 0.95) with hemoglobin can introduce multicollinearity issues, compromising result accuracy. Hemoglobin, providing a direct and clinically meaningful measure of oxygen-carrying capacity, was chosen over hematocrit due to clinical and practical considerations.

**Table 1:** Association between patient characteristics and the need for packed red blood cell transfusion in cardiac surgery patients.

Variables	Overall	Packed red blood cells		*P*-value
		None (n=211)	One or more (n=284)	
Age (years), median (SD)	56.66 (14.17)	55.35 (12.55)	57.63 (15.22)	0.011^MW*^
Body surface area (m^2^), mean (SD)	1.74 (0.21)	1.79 (0.21)	1.71 (0.20)	< 0.01^t^^*^
Hematocrit, mean (SD)	33.9 (6.50)	36.00 (6.49)	32.36 (6.07)	< 0.01^t^^*^
Hemoglobin (%), mean (SD)	11.3 (2.17)	12.07 (2.17)	10.76 (1.99)	< 0.01^t^^*^
Creatinine (mg/dl), median (SD)	1.18 (0.91)	1.10 (0.82)	1.23 (0.97)	0.57^MW^
Sex				< 0.001^c*^
Male	299 (60.40%)	149 (49.83%)	150 (50.17%)	
Female	196 (39.60%)	62 (31.63%)	134 (68.37%)	
Diabetes mellitus				0.934^c^
No	347 (70.10%)	147 (42.36%)	200 (57.64%)	
Yes	148 (29.90%)	64 (43.24%)	84 (56.76%)	
High blood pressure				0.335^c^
No	170 (34.34%)	78 (45.88%)	92 (54.12%)	
Yes	325 (65.66%)	133 (40.92%)	192 (59.08%)	
Prior cardiac surgery				0.002^c^
No	459 (92.73%)	205 (44.66%)	254 (55.34%)	
Yes	36 (7.27%)	6 (16.67%)	30 (83.33%)	
CPB				0.003^c^
No	15 (3.03%)	12 (5.69%)	3 (1.06%)	
Yes	480 (96.97%)	199 (94.31%)	281 (98.94%)	
Urgency				0.057^c^
No	441 (89.09%)	195 (44.22%)	16 (29.63%)	
Yes	54 (10.91%)	246 (55.78%)	38 (70.37%)	
Type of surgery				0.353^F^
Aortic surgery	29 (5.9%)	12 (41.4%)	17 (58.7%)	
CABG	207 (41.8%)	88 (42.5%)	119 (57.5%)	
Combined	25 (5.1%)	6 (24%)	19 (76%)	
Valve	183 (37%)	84 (45.9%)	99 (54.1%)	
Others	51 (10.3%)	21 (41.2%)	30 (58.8%)	

MW Mann-Whitney U test

*^t^*unpaired *t*-test

^c^Pearson’s Chi-square test

^F^Fisher’s exact test

^*^Statistically significant (*P*<0.05)

CABG=coronary artery bypass grafting; CPB=cardiopulmonary bypass; SD=standard deviation

The results of the ML models compared to TRACK and TRUST are presented in Table 2. The LR, SVM, and MLP models exhibited comparable accuracy scores, ranging from 0.6714 to 0.6719. However, RF, TRACK, and TRUST displayed slightly lower accuracy. Regarding precision, SVM, LR, and RF demonstrated similar performance, while TRACK, MLP, and TRUST showed slightly lower precision. MLP and TRUST demonstrated superior performance in terms of recall, exhibiting higher average values. Notably, TRUST achieved the highest recall among all the models, albeit with a notable standard deviation, indicating substantial variability in sensitivity across different runs. Evaluating the F1 score, the ML models achieved similar results, ranging from 0.6926 to 0.7325, while TRACK and TRUST exhibited slightly lower F1 scores. Furthermore, in terms of area under the curve (AUC), the ML models displayed comparable performance, ranging from 0.6622 to 0.7350, while TRACK and TRUST demonstrated slightly lower AUC scores.

**Table 2:** Summary of model performance metrics.

Metric model	Accuracy	Precision	Recall	F1	AUC
LR	0.6719 ± 0.0530	0.7196 ± 0.0499	0.7058 ± 0.0711	0.7106 ± 0.0492	0.7350 ± 0.0511
MLP	0.6714 ± 0.0479	0.6883 ± 0.0447	0.7896 ± 0.0820	0.7325 ± 0.0430	0.7333 ± 0.0515
RF	0.6588 ± 0.0470	0.7162 ± 0.0460	0.6750 ± 0.0740	0.6926 ± 0.0489	0.7079 ± 0.0545
SVM	0.6717 ± 0.0482	0.7196 ± 0.0475	0.7049 ± 0.0654	0.7103 ± 0.0451	0.7324 ± 0.0493
TRACK	0.6278 ± 0.0470	0.7061 ± 0.0511	0.6049 ± 0.0672	0.6495 ± 0.0503	0.6757 ± 0.0518
TRUST	0.6189 ± 0.0526	0.6491 ± 0.0459	0.7494 ± 0.1453	0.6840 ± 0.0890	0.6622 ± 0.0519

AUC=area under the curve; LR=logistic regression; MLP=multi-layer perceptron; RF=random forest; SVM=support vector machine; TRACK=Transfusion Risk and Clinical Knowledge; TRUST=Transfusion Risk Understanding Scoring Tool

The AUC is widely acknowledged as a robust metric for evaluating binary classification problems. It captures the capacity of the models to differentiate between positive and negative instances across various probability thresholds, encompassing both sensitivity and specificity. AUC offers several advantages, including resilience to class imbalance, independence from decision thresholds, and the ability to provide an overall measure of discriminative power. Moving to the statistical test results, Table 3 presents the comparisons among LR, MLP, RF, SVM, TRACK, and TRUST models based on the AUC metric. The table displays the *P*-values for pairwise comparisons, using a significance level of 0.05.

**Table 3:** Statistical test results for area under the curve metric.

LR		0.49	< 0.05	0.11	< 0.05	< 0.05
MLP			< 0.05	0.47	< 0.05	< 0.05
RF				< 0.05	< 0.05	< 0.05
SVM					< 0.05	< 0.05
TRACK						0.14
TRUST						
	LR	MLP	RF	SVM	TRACK	TRUST

LR=logistic regression; MLP=multi-layer perceptron; RF=random forest; SVM=support vector machine; TRACK=Transfusion Risk and Clinical Knowledge; TRUST=Transfusion Risk Understanding Scoring Tool

All ML models, including LR, MLP, RF, and SVM, demonstrated statistical superiority over the TRACK and TRUST models, as evident from the statistical test results. Among these ML models, LR exhibited the highest AUC score, which was found to be statistically equivalent to the AUC scores of MLP and SVM. The choice of LR as the preferred model can be justified by its simplicity compared to MLP and SVM. LR is a linear model that offers straightforward interpretability and requires fewer computational resources, making it a practical choice for many applications. While MLP and SVM may provide more complex modeling capabilities, the added complexity may not necessarily lead to significant performance gains in terms of AUC. Therefore, considering the comparable performance and the simplicity of the LR model, it emerges as a favorable choice for the given task.

**Fig. 1:**
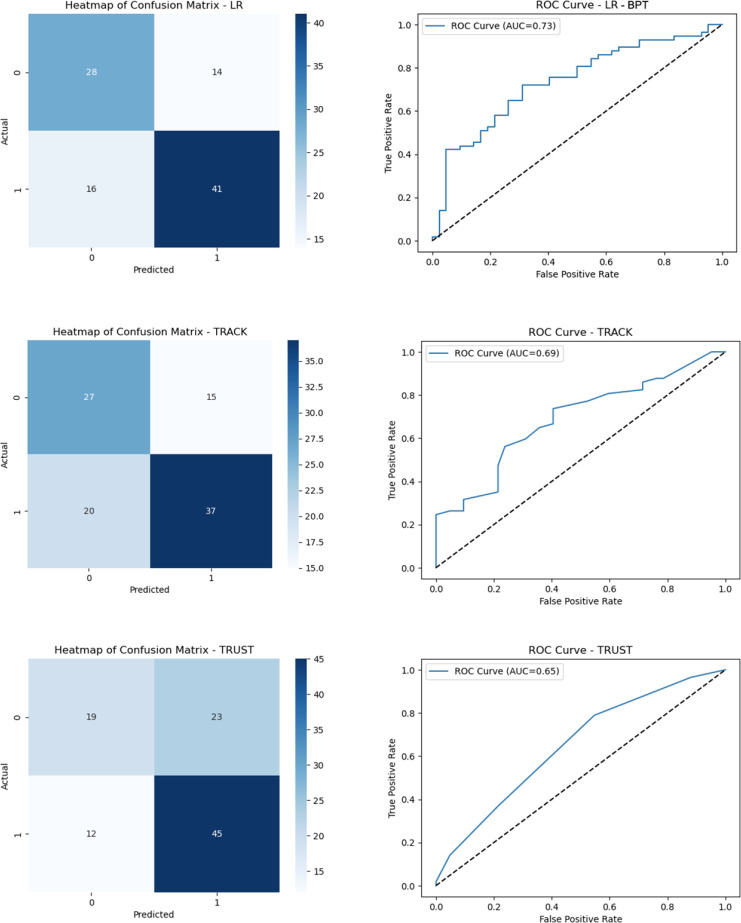
Performance comparison of logistic regression (LR), Transfusion Risk and Clinical Knowledge (TRACK), and Transfusion Risk Understanding Scoring Tool (TRUST) models on test data: confusion matrix and receiver operating characteristic (ROC) curve analysis. AUC=area under the curve; BPT=blood prediction tool.

Figure 1 presents the performance of the BPT (using LR), TRACK, and TRUST models on the test data, showcasing their confusion matrix and receiver operating characteristic (ROC) curve. The confusion matrix provides insights into the true positives, false positives, true negatives, and false negatives, while the ROC curve illustrates the trade-off between the true positive rate and false positive rate. Among the models, LR outperformed the others with an AUC of 0.71, followed by TRACK and TRUST with AUCs of 0.68 and 0.66, respectively. It is evident that LR exhibited superior sensitivity and precision compared to TRACK and TRUST. The LR model’s confusion matrix revealed a higher count of true positives and true negatives, indicating its proficiency in correctly identifying positive and negative cases. Conversely, both TRACK and TRUST demonstrated relatively higher rates of false positives and false negatives, underscoring the LR model’s effectiveness in accurately classifying the test data.

**Fig. 2:**
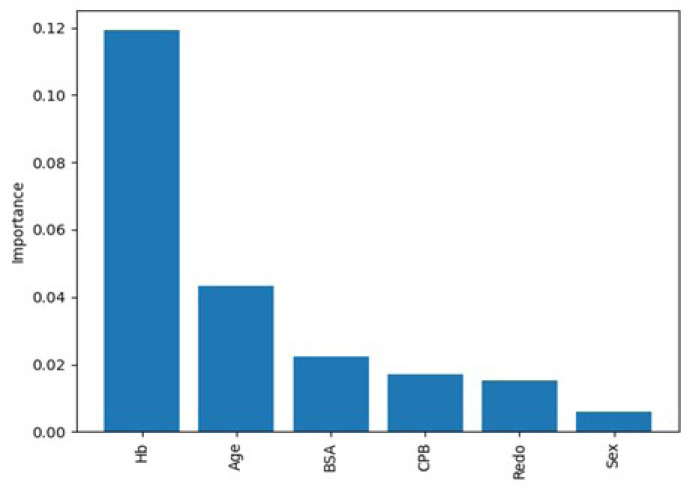
Visualization of feature importance ranking using permutation importance. Hb=hemoglobin; BSA=body surface area; CPB=cardiopulmonary bypass.

The PI technique was employed to assess the relative importance of features in the predictive model. The resulting bar chart in Figure 2 visually represents the descending order of feature importance. By permuting the values of each feature and observing the resulting impact on model performance, valuable insights were obtained regarding the influence of features on the model’s predictions. Hemoglobin emerged as the feature with the highest PI, indicating its significant influence on the model’s predictions. Age demonstrated moderate importance, while BSA and CPB exhibited comparatively lower but still notable influence. On the other hand, redo surgeries and sex had relatively lesser impacts on the model’s predictions.

**Fig. 3:**
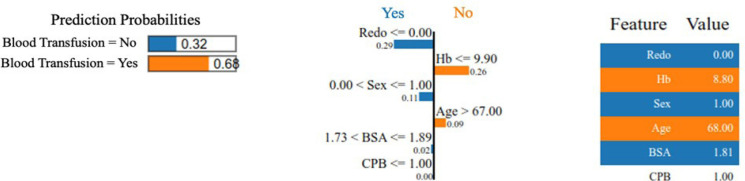
Application of the local interpretable model-agnostic explanations (or LIME) technique for local interpretability. Hb=hemoglobin; BSA=body surface area; CPB=cardiopulmonary bypass.

The Figure 3 exemplifies the application of the LIME technique to a specific instance, providing insights into how the tool considers all the features to make a prediction.

## DISCUSSION

During and following the coronavirus disease 2019 (COVID-19) era, blood donation has become increasingly challenging. Disturbingly, studies have indicated a significant decline in donation rates, with some states in Brazil experiencing a reduction of up to 38%, leading to reports of blood centers facing critical shortages^[15]^. Moreover, existing research has consistently linked blood transfusions to adverse outcomes, including heightened morbidity and mortality rates^[1,2]^. Given this concerning backdrop, it becomes crucial to identify individuals who are at a higher risk of requiring red cell transfusions. By doing so, it becomes possible to implement preventive and supportive measures, effectively mitigating the associated risks and enhancing patient safety in the context of blood transfusions.

Risk predictor tools have emerged as a modern approach to effectively manage risks and allocate resources. Notably, a systematic review revealed the publication of 169 prediction tools utilizing artificial intelligence during the COVID-19 pandemic, highlighting the growing interest in this area^[16]^. However, despite the existence of globally utilized risk prediction scores for blood transfusion in cardiac surgery, their validation in the Brazilian population remains insufficient^[7]^. Several factors have been proposed to explain this discrepancy, ranging from the unique characteristics of the Brazilian population as a developing country, where anemia prevails at higher rates compared to developed nations, to the limited access of Brazilian patients to globally employed equipment for intraoperative blood reuse. Importantly, the lack of cost-effectiveness and absence of coverage by the public health system (Sistema Único de Saúde or SUS) have hindered the adoption of such devices in Brazil^[17]^.

This study aimed to develop a practical and reliable risk score consisting of variables that can be easily utilized at the bedside. The performance of the developed score, as measured by AUC, was found to be comparable to the internal validation results of two commonly used risk scores in the healthcare field: TRUST (AUC = 0.79) and TRACK (AUC = 0.73). It is noteworthy that the BPT, which incorporates variables such as hemoglobin level, BSA, sex, age, use of CPB, and redo surgery, shares significant similarities with the features employed in TRUST (hemoglobin level, weight, sex, age, nonelective surgery, creatinine level, redo, nonisolated surgery) and TRACK (age, weight, sex, hematocrit, and complex surgery). However, the distinction lies in the specific patients’ characteristics on which they are based, and the calculation methods used for prediction. Also, unlike other tools, BPT was developed using ML.

It is true that it has been showed ML not being superior to traditional LR, especially in small samples like the presented in this study. However, because of its ability of constantly improve its predictive value as it is exposed to new data, starting with a reasonable accuracy at baseline, it might become a better model in the long run^[18]^.

Hemoglobin levels have been established as a significant prognostic factor for transfusion requirements, carrying substantial scientific evidence. Numerous studies have consistently revealed a direct correlation between lower preoperative hemoglobin levels and an elevated probability of necessitating transfusions, while conversely, higher hemoglobin levels are associated with a decreased risk^[3,4,7]^. These findings, supported by multiple investigations, emphasize the criticality of diligent monitoring and effective management of hemoglobin levels both before and during surgical interventions as a fundamental approach to diminish transfusion needs^[19]^.

An interesting aspect contributing to the failure of international prediction tools in accurately anticipating blood transfusion requirements within the Brazilian population can be attributed to the pronounced disparity in hemoglobin levels between Brazil and developed nations. Specifically, extensive research has highlighted that the hemoglobin level in the Brazilian population is considerably lower compared to that observed in more developed countries. Consequently, it becomes imperative to account for this distinction when adapting and applying prediction tools within the Brazilian healthcare context to ensure their efficacy and relevance.

BSA has also been identified as an important predictor of transfusion requirements during cardiac surgery. Several studies have shown that patients with a smaller BSA are more likely to require transfusions compared to those with a larger BSA^[3,6]^. This relationship can be explained by the fact that patients with a smaller BSA may have a smaller blood volume, which makes them more susceptible to blood loss during surgery. Moreover, these patients are more affected by the hemodilution used in CPB^[20]^. Therefore, taking BSA into account when predicting transfusion requirements can help identify high-risk patients and optimize blood management strategies, including maneuvers to decrease hemodilution in CPB^[20,21]^.

Sex and age are other important predictors of transfusion requirements during cardiac surgery. Several studies have shown that female patients are more likely to require transfusions compared to male patients^[3,4]^. Although female sex has been associated to increase bleeding in several surgical analyses, the reason is still under debate. This increased hazard of bleeding has been theorized to be due to smaller BSA, increased frailty, and sex hormone differences^[22,23]^. Age has also been identified as an important predictor, with older patients being more likely to require transfusions^[3,4]^. This can be explained by the fact that older patients have increased frailty and are more susceptible to blood loss during surgery^[24]^. Therefore, sex and age should be considered when predicting transfusion requirements and developing blood management strategies.

Use of CPB was also another factor considered important for prediction by the tool. CPB has characteristics intrinsic to its use, such as hemodilution, heparinization, and consumption of coagulation factors and platelets, which predispose to an increased risk of bleeding and a decrease in serum hemoglobin levels^[25]^. However, there are several maneuvers that can be done in order to try to minimalize this risk, like matching the size of the CPB circuit to the size of the patient, autologous priming of CPB circuit, including retrograde arterial and venous antegrade priming, and perioperative blood cell recovery and reinfusion^[20]^.

### Limitations

This study had several limitations that should be acknowledged. Firstly, the data used in this study was obtained from a single center located in northeast Brazil, which may limit the generalizability of the findings to other populations or regions. Additionally, while the dataset of 500 patients may appear substantial, it is important to note that ML algorithms tend to perform better with larger datasets. Recognizing this, our research group is currently working on a project for multicentric validation and calibration of the tool, with the aim of enhancing its reliability and applicability across different settings.

Furthermore, it is important to acknowledge that this study did not consider other variables that could potentially contribute to increased surgical bleeding, such as coagulopathy or the use of anticoagulant medications. Additionally, the study did not consider the use of other blood products, such as frozen plasma, platelets, or cryoprecipitates, which may also impact bleeding outcomes. These factors should be considered in future research to provide a more comprehensive understanding of the predictors of surgical bleeding.

## CONCLUSION

The blood transfusion prediction tool, BPT, was developed for application in patients undergoing major cardiac surgery. In comparison to other widely used tools available globally, BPT demonstrated superior accuracy while maintaining a user-friendly interface with only six variables. Furthermore, BPT holds the potential for calibration and refinement over time, ensuring its continued relevance and effectiveness.
