# ERRATUM

**DOI:** 10.21470/1678-9741-2023-0212e

**Published:** 2024-05-13

In the article “Predicting the Need for Blood Transfusions in Cardiac Surgery: A Comparison between Machine Learning Algorithms and Established Risk Scores in the Brazilian Population?”, with DOI code https://doi.org/10.21470/1678-9741-2023-0212, published in the Brazilian Journal of Cardiovascular Surgery, 39.2, the surnames of the fourth, fifth and sixth authors are misspelled:

In the original version the information was: Cristiano Berardo Carneiro da Cunha^1,2,3^, MD, MSc, PhD; Tiago Andrade Lima^4^, PhD; Diogo Luiz de Magalhães Ferraz^3^, MD; Igor Tiago Correia Silva^3^, MD; Matheus Kennedy Dionisio Santiago^4^, KDS.; Gabrielle Ribeiro Sena^5^, MD, PhD; Verônica Soares Monteiro^6^, MD, PhD; Lívia Barbosa Andrade^7^, PT, PhD

The correct information is: Cristiano Berardo Carneiro da Cunha^1,2,3^, MD, MSc, PhD; Tiago Pessoa Ferreira de Lima^4^, PhD; Diogo Luiz de Magalhães Ferraz^3^, MD; Igor Tiago Correia Silva^3^, MD; Matheus Kennedy Dionisio Santiago^4^, KDS.; Gabrielle Ribeiro Sena^5^, MD, PhD; Verônica Soares Monteiro^6^, MD, PhD; Lívia Barbosa Andrade^7^, PT, PhD

